# Fuke Qianjin Combined with Antibiotic Therapy for Pelvic Inflammatory Disease: A Systematic Review and Meta-Analysis

**DOI:** 10.1155/2020/5372839

**Published:** 2020-07-21

**Authors:** Yu Chen, Shaobin Wei, Li Huang, Mei Luo, Yang Wu, Caimmiao Yin

**Affiliations:** ^1^Department of Gynecology, Hospital of Chengdu University of Traditional Chinese Medicine, Chengdu 610072, Sichuan, China; ^2^Department of Gynecology, Hospital of Chongqing Institute of Traditional Chinese Medicine, Chongqing 400011, China

## Abstract

**Background:**

Pelvic inflammatory disease (PID) without timely and proper treatment can cause long-term sequelae; meanwhile, patients will be confronted with the antimicrobial resistance and side effects. Chinese patent medicine as a supplement is used to treat PID with satisfactory clinical efficacy. This study evaluated the efficacy and safety of Fuke Qianjin (FKQJ) combined with antibiotics in the treatment of PID.

**Methods:**

Eight electronic databases and other resources were searched to make a collection of the randomized controlled trials (RCTs) from 1990 to 2019. The RCTs contrasting the effect of FKQJ combined with antibiotics regimens and antibiotics alone in reproductive women with PID were included. The antibiotics regimens are all recommended by the guidelines. Two reviewers independently screened the studies, extracted the data, and assessed the methodological quality of the included studies. Then, the meta-analyses were performed by RevMan 5. 3 software if appropriate.

**Results:**

Twenty-three RCTs (2527 women) were included in this review. The evidence showed that FKQJ combined with antibiotics improved the markedly effective rate compared to antibiotics alone group (RR = 1.38, 95% CI 1.27 to 1.49, *I*^2^ = 42%), shortened the improvement time of low abdominal pain (MD = −1.11, 95% CI −1.39 to −0.84, *I*^2^ = 38%), and increased the rate of lower abdominal pain improvement (RR = 1.35, 95% CI 1.19 to 1.55, *I*^2^ = 0). The implementation of adjuvant reduced the recurrent rate compared with antibiotics alone (RR = 0.27, 95% CI 0.13 to 0.56, *I*^2^ = 0%).

**Conclusions:**

Based on available evidence, FKQJ combined with antibiotics therapy have certain outcomes on increasing the markedly effective rate, decreasing the recurrent rate compared with antibiotics alone group. This therapy appears to improve lower abdominal pain and curtail the relief time. Due to the low quality and the risk of bias, any high-quality evidence or longer follow-up period should be advisable and necessary in the future.

## 1. Introduction

### 1.1. Background

PID is a spectrum of inflammatory disorders of the upper female genital tract, including endometritis, salpingitis, tubo-ovarian abscess, and pelvic peritonitis [1]. Without timely and thorough treatment PID can cause a series of sequelae including chronic pelvic pain, pelvic adhesion, and infertility [2]. A population-based nested controlled study, which included 18,276 women with a new diagnosis of infertility and 73,104 matched controls, showed PID were associated with an increased risk of infertility in women aged ≤40 years [3]. Reproductive tract infection, especially *N. gonorrhoeae* and *C. trachomatis* [4], and uterine cavity operation are the most common causes of PID. The age is mainly concentrated from 25 to 35, and more than 1% of sexually mature females are estimated to be suffering from PID [5]. In developing countries, the incidence of PID among women of childbearing age is 40% [6]. It is the most common gynecological cause for hospital admission in America, and in England, even though the incidence is decreasing, 1.1% of young women attending primary care services are diagnosed with PID [7–9]. Although there is a reduction in the rate of hospitalisation in developed countries, PID is still the most common gynecological disease, and over 1% of young women are diagnosed with PID when attending primary care services [7].

As the guideline of the US Centers for Disease Control and Prevention (CDC) suggests, the treatment of PID is ordinarily with broad-spectrum antibiotics [10]. However, due to the inappropriate and irrational use of antibiotics, there are problems of drug-resistant bacteria, reduced drug sensitivity, and some side effects. Traditional Chinese medicine (TCM) has a long-standing history, with exact efficacy and obvious advantages in treating gynecological diseases. The evidence suggests that Chinese patent medicine combined with antibiotics in the treatment of PID has a remarkable curative effect, improves the clinical symptoms effectively, and reduces the relapse rate notably [11]. Fuke Qianjin tablets/capsules (FKQJ) are a pure traditional Chinese medicine, used in the treatment of PID. The principal constituents of FKQJ are Philippine flemingia root, Cherokee rose root, *Andrographis paniculata* Nees (APN), Leatherleaf mahonia, *Zanthoxylum dissitum* Hemsl, *Angelica sinensis*, Lignum millettiae, and *Codonopsis pilosula*. The main therapeutic effects of FKQJ are clearing heat and dehumidifying and benefiting qi and stasis. APN, one of FKQJ's main components, has been reported to have antioxidant [12] and immunomodulatory effects [13], and likewise it showed a potent anti-inflammatory effect on pathogen-induced PID in rats [14]. Although there was a meta-analysis about the treatment of PID with FKQJ combined with antibiotics and some with TCM, the optimal treatment strategy and the safety were still controversial. And more than ten related RCTs have been published in the past two years. Therefore, this systematic review and meta-analysis of RCTs about FKQJ combined with antibiotics for the treatment of PID were conducted to conclude a comprehensive assessment of FKQJ as an adjunctive therapy of PID.

## 2. Method

### 2.1. Study Registration

The registration number of this systematic review on PROSPERO is CRD42019131527.

### 2.2. Search Strategy

We searched the Cochrane Central Register of Controlled Trials, PubMed, Embase, Medline, and four Chinese databases–the China National Knowledge Infrastructure Database (CNKI), the Wanfang Database, the China Science and Technology Journal Database (VIP), and the Chinese Biology Medicine (CBM)—to make a collection of the RCTs about FKQJ combined with antibiotic therapy from 1990 to December 2019. And we also searched clinical trials in progress from the NIH Clinical Trials, the International Clinical Trials, and the Chinese Clinical Register. The language was limited to Chinese and English. We used the following search terms ((pelvic inflammatory disease OR PID OR endometritis OR salpingitis) AND (fukeqianjin OR fuke qianjin OR Chinese patent medicine) NOT sequelae NOT chronic). The titles and abstracts of all literature were reviewed and investigated to eliminate repetitive or irrelevant articles.

### 2.3. Eligibility Criteria and Study Selection

Two reviewers evaluated potentially relevant RCTs independently by reading the whole article based on the inclusion criteria. Disagreements about inclusion and exclusion were resolved by consensus or consulting the third reviewer. The following inclusion criteria were the eligibility criteria for the study selection.

#### 2.3.1. Types of Studies

Data from RCTs and quasi-randomized trials were sought electronically. The RCTs comparing FKQJ combined with antibiotic therapy for PID were included.

#### 2.3.2. Types of Participants

They are patients of childbearing age diagnosed with acute PID by clinical symptoms or auxiliary examinations based on the criteria of guidelines and teaching materials. Patients who are systemically unwell, with presence of a tubo-ovarian abscess, who are pregnant, undergoing surgery, or after salpingography were excluded.

#### 2.3.3. Types of Interventions

We limited antibiotics without any dosage form restriction for the control group, which were all recommended by the 2015 US CDC guidelines for PID [10]. And we did not limit the dosage forms of FKQJ as orally ingested product (such as capsules and tablets).

#### 2.3.4. Types of Outcome Measures

Primary outcomes were the rate of markedly effective and adverse events. Secondary outcomes were the symptom improvement of leucorrhea, the improvement of lower abdominal pain, the recurrent rate at half year, recurrent rate, and the time of abdominal pain disappearance.

### 2.4. Assessment of Risk of Bias

Two reviewers independently evaluated the risk of bias for the included studies using the Cochrane Collaboration's tool for evaluating the risk of bias [15], consensus, or consulting with the third author if necessary. This tool supports the consideration of sequence generation, allocation concealment, blinding, incomplete outcome data, selective outcome reporting, and other sources of bias. We assessed the risk of bias in each source as low risk of bias, high risk of bias, and unclear risk of bias and contacted the study's authors to request for missing information by using open-ended questions if necessary.

### 2.5. Data Extraction and Management

The authors independently extracted the data from the included studies by a standardised Excel form. We collected the following data: author, year, country, sample size, age, the dosage of FKQJ, the dosage, and regimen of antibiotics. We checked the data for erratum, retraction, fraud, and inconsistencies. If a study had more than two intervention arms, we included or combined only those that met the predefined inclusion criteria.

### 2.6. Statistical Analysis

We extracted both dichotomous and continuous data. For dichotomous data, the number of events in each group was used to calculate risk ratios (RR), with all outcomes presenting 95% confidence intervals (CI). For continuous data, we calculate the mean difference (MD), with all outcomes presenting 95% confidence intervals (CI). Subgroup analysis was examined according to the route of antibiotic administration and the length of therapy. Heterogeneity between trials and subgroups of trials with different regimens of antibiotics was calculated using chi-square statistic and analysed with the *I*^2^ statistics. If the substantial heterogeneity (*I*^2^ 50% or higher) was identified, we used a random-effects analysis. For each outcome with more than ten studies, the publication bias was examined by the funnel plot.

## 3. Results

### 3.1. Description of Studies

#### 3.1.1. Results of the Search

**Figure 1:**
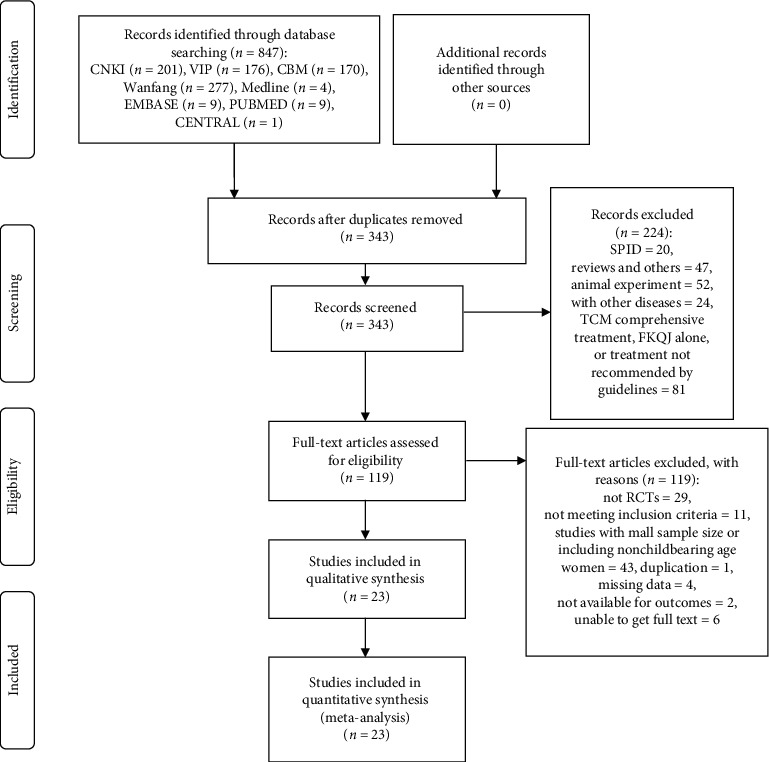
Flow diagram of the literature search process based on the PRISMA guideline. PRISMA: Preferred Reporting Items for Systematic Reviews and Meta-Analyses; CNKI: China National Knowledge Infrastructure Database; VIP: the China Science and Technology Journal Database; CBM: Chinese Biology Medicine (CBM); RCT: randomized controlled trials.

The search and selection process of this review was described based on the Preferred Reporting Items for Systematic Reviews and Meta-Analyses (PRISMA) guideline [16] in Figure 1. We searched the CENTRAL, PubMed, Embase, Medline, CNKI, the Wanfang Database, VIP, and CBM and provided a total of 847 references. After eliminating the duplicate literature, 343 records remained. Of these, 224 records were discarded as clearly irrelevant and we considered 119 full-text studies. After full-text review, 23 articles met our inclusion criteria, and we excluded 96 studies. The most common reason for exclusion was that these studies were with small sample size or included nonchildbearing age women (43 studies). Other common reasons for exclusion were as follows: the therapeutic regimen did not meet the inclusion criteria (11 studies), studies without detailed information (4 studies), or nonrandomized studies (29 studies) (Figure 1). Finally, 23 RCTs (2527 women) were identified for inclusion in the review (Table 1).

## 4. Risk of Bias in Included Studies

**Figure 2:**
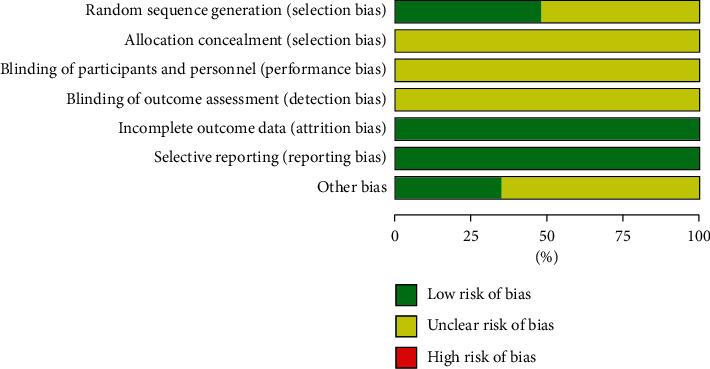
Risk of bias.

**Figure 3:**
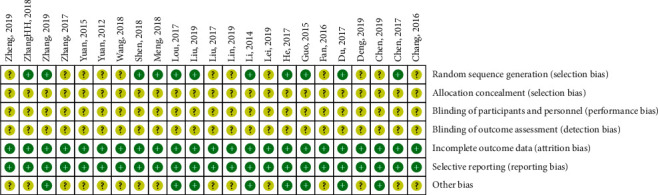
Summary risk of bias graph. *Note*. “+”: low risk of bias; “?”: unclear risk of bias.

We assessed the risk of bias of the 23 included trials in Figures 2 and 3. Ten trials mentioned the way of generating the allocation sequences. Ten trials [17–19, 24, 25, 27–29, 32, 37] used the random number table, and Meng [20] used a computer random number generator. All included trials did not mention blinding of participants and personnel or the blinding of outcome assessment, which makes the risk of performance and detection bias unclear. The publication bias was assessed through the funnel plot for comparison when there were 10 or more studies in the same analysis.

## 5. Synthesis of Results

We analysed the outcomes in five subgroups based on the different regimens of antibiotics class comparison:

- Regimen A containing FKQJ combined with nitroimidazoles versus nitroimidazoles
- Regimen B containing FKQJ combined with clindamycin versus clindamycin
- Regimen C containing FKQJ combined with quinolones versus quinolones
- Regimen D containing FKQJ combined with cephalosporin plus nitroimidazoles versus cephalosporin plus nitroimidazoles
- Regimen E containing FKQJ combined with cephalosporin plus doxycycline versus cephalosporin plus doxycycline

### 5.1. Primary Outcomes

#### 5.1.1. Markedly Effective Rate

**Figure 4:**
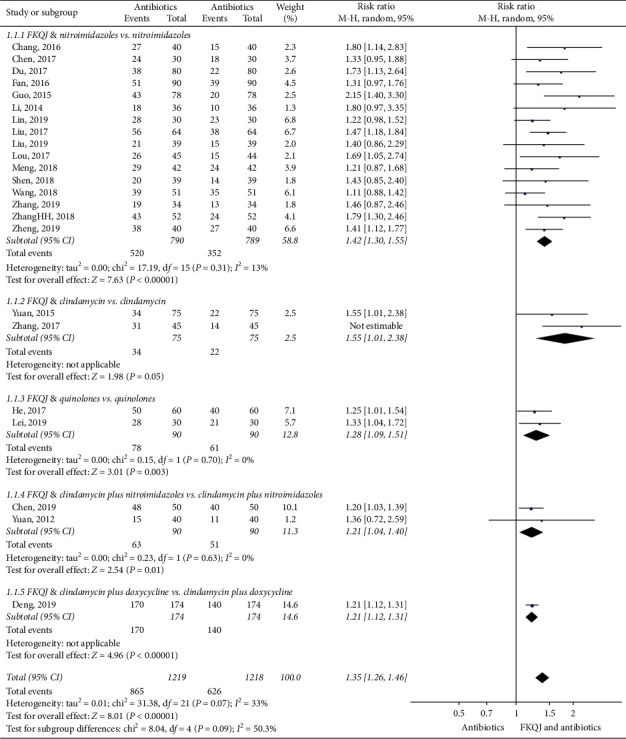
Forest plot—markedly effective rate; FKQJ, Fuke Qianjin.

**Figure 5:**
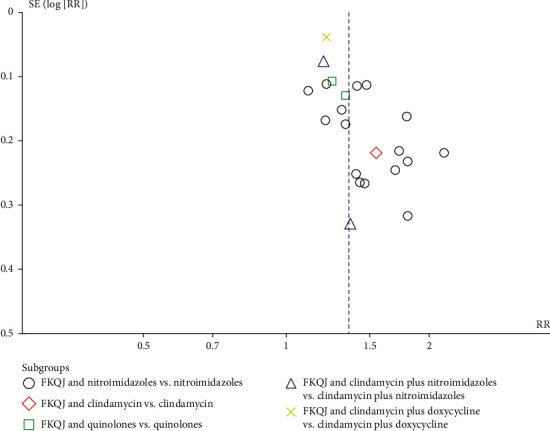
Funnel plot—markedly effective; FKQJ, Fuke Qianjin.

The calculation of markedly effective rate is the sum number of cured cases and effective cases divided by the total number of cases. All inclusion studies with a total of 2527 patients reported the markedly effective rate in results. With the heterogeneity for this analysis, a random-effect model was conducted to estimate pooled effect size. Meanwhile, based on the different antibiotics regimens, subgroup analysis was performed. The results indicated that FKQJ combined with antibiotics improved the effective rate compared with antibiotics alone (*P* < 0.00001) (RR = 1.38, 95% CI 1.27 to 1.49, *I*^2^ = 42%) (Figure 4). The subgroup analysis indicated difference (*P*=0.03). The asymmetrical funnel plot observed potential publication bias (Figure 5).

Because of the high heterogeneity, a sensitivity analysis was performed. When we removed the trial, Zhang [33], in sensitivity analyses of the markedly effective rate, there was an impact on the random-effects pooled efficacy difference (*P*=0.07) (RR = 1.35, 95% CI 1.26 to 1.46, *I*^2^ = 33%) and the subgroup analysis suggested no difference (*P*=0.09).

#### 5.1.2. Adverse Events

Two studies [25, 26] mentioned adverse events. Wang et al. [26] reported no adverse events, and Shen [25] reported adverse events and provided data in results. The FKQJ combined with antibiotics group [5.13%, (2/39)] did not increase the rate of adverse events compared with antibiotics alone group [5.13%, (2/39)]. The adverse reactions were slight nausea, vomiting, or dizziness, and all adverse reactions can be alleviated or eliminated after symptomatic treatment.

### 5.2. Secondary Outcomes

#### 5.2.1. Disappearance Time of Lower Abdominal Pain

**Figure 6:**
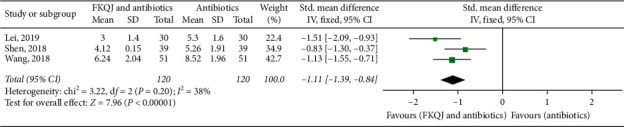
Forest plot—time of lower abdominal pain disappearance; FKQJ, Fuke Qianjin.

Three studies [25, 26, 38] reported the disappearance time of lower abdominal pain in results. The FKQJ combined with antibiotics group significantly shortened the length of lower abdominal pain disappearance (*P* < 0.00001) (MD = −1.11, 95% CI −1.39 to −0.84, *I*^2^ = 38%) (Figure 6).

#### 5.2.2. Improvement of Lower Abdominal Pain

**Figure 7:**
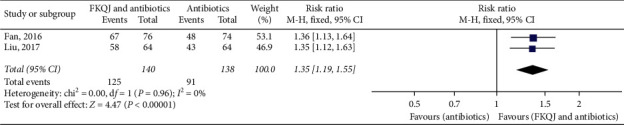
Forest plot—improvement of lower abdominal pain. FKQJ, Fuke Qianjin.

Two trials [22, 23] mentioned the improvement of lower abdominal pain. The analyses showed the experimental group had a higher symptom improvement rate (*P* < 0.00001) (RR = 1.35, 95% CI 1.19 to 1.55, *I*^2^ = 0) (Figure 7).

#### 5.2.3. Recurrent Rate

**Figure 8:**
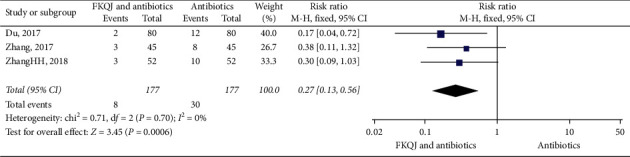
Forest plot—comparison for recurrent rate. FKQJ, Fuke Qianjin.

The recurrence rate in half a year was reported in three trials [18, 19, 33]. The implementation of adjuvant FKQJ reduced the recurrent rate compared with antibiotics alone (*P*=0.0006) (RR = 0.27, 95% CI 0.13 to 0.56, *I*^2^ = 0%) (Figure 8).

#### 5.2.4. Improvement of Leucorrhea

Only one trial [23] reported the improvement rate of leucorrhea. The FKQJ group had a greater improvement of leucorrhea [90.63%, 58/64] than the control group [65.63%, 42/64]

## 6. Discussion

This study systematically reviewed the efficacy and safety of FKQJ combined with antibiotics in the treatment of PID on the basis of existing literature and data. There was only one meta-analysis [40] of adjuvant FKQJ for PID in 2016. That meta-analysis only included sixteen trials of FKQJ combined with antibiotics in the treatment of endometritis from 2010 to 2016. However, that study focused on outcomes related to efficacy, such as the thickness of the endometrium, the occurrence rate of the normal menstrual cycle, and total effective rate, and safety-related outcomes were not available. Furthermore, more than 20 articles have been published in the past two years. Comparing with the previous study, this study investigated not only the efficacy but also the safety evaluation.

Chinese material medicine is an integral part of TCM, with thousands of years of clinical application history. Chinese patent medicines mainly come from ancient classical prescriptions and current clinical effective prescriptions. As recommended in the Chinese Guidelines for PID, Chinese material medicine and Chinese patent medicine were conventionally used in the treatment of PID [41]. To standardise the application of Chinese patent medicine in the treatment of PID, the Chinese Association of Chinese Medicine (CACM) promulgated *the Clinical Practice Guideline on Traditional Chinese Medicine Alone or Combined with Antibiotics for Patients with Pelvic Inflammatory Disease* in 2017. A metabolomics study revealed that FKQJ had the efficacy of potential therapeutic to multiple pathogens induced PID by reducing inflammation and improving metabolic disorders [42]. Although the adjuvant treatment of PID with traditional Chinese medicine has clinical efficacy, its mechanism and safety are also worthy of attention.

This meta-analysis of twenty-three studies involved 2527 participants showing that FKQJ combined with antibiotics had advantages over antibiotics alone in improving the markedly effective rate. Auxiliary use of proprietary Chinese medicines did not lead to adverse events. We seemingly found the marked efficacy of FKQJ combined with antibiotics for PID had a certain correlation with the difference of antibiotics regimen. However, because of the less information about the pathogen culture in participants, it is difficult to analyse which regimen is better. The results showed that the clinical recurrence rate tends to decrease. We considered that FKQJ combined with antibiotics could reduce the clinical recurrence rate of PID according to the existing evidence. However, this conclusion needed more high-quality and substantial study sample evidence to support in the future. We consider the natural pregnancy rate as an outcome in the protocol, but after abstraction there was no data on the rate of fertility. Therefore, it is necessary to extend the follow-up time in future clinical research.

Nevertheless, our meta-analysis exhibited several limitations. Firstly, the quality of evidence was low in some comparisons because of the unclear or high risk of bias. The heterogeneity of most subgroups was acceptable, yet within some subgroups it remained high (e.g., difference of the treatment courses). Secondly, the FKQJ formulae included in this study were mainly Chinese herbal compound capsules and tablets, and the dosages, the forms, and manufacturer information were various. Although we judged the rationality of FKQJ use in each trial according to the medicine instructions, to our minds, there still was heterogeneity in interventions between the included studies. Furthermore, the regimens and administrations of antibiotics varied greatly across trials. According to the guideline, the parenteral regimens appeared to have similar efficacy to oral regimens in women with PID. Therefore, the types of antibiotics provide the basis for ultimate subgroup analysis. Fourthly, the funnel plot is not symmetrical, and there may be publication bias. Additionally, only one trial of 78 participants provided data about adverse events, and all RCTs did not report the rate of fertility after treatment. Due to the inadequate sample size and data, it is shown that few trials were contributing to some outcomes.

## 7. Conclusions

FKQJ combine with antibiotics therapy have effects on improving the markedly effective rate and reducing the recurrence rate. This therapy appears to improve lower abdominal pain and curtail the relief time in patients after treatment. Antibiotics plus Chinese patent medicine did not increase the incidence of adverse events. However, due to the poor methodological quality and the high heterogeneity of some included trials, our conclusions should be carefully interpreted. To assess the pregnancy rate and safety evaluation after the standardised treatment, future trials based on high-quality evidence should perform a longer period of follow-up for 12 months or more to conduct the convincing conclusions in the future.

## Figures and Tables

**Table 1:** Characteristics of included studies.

First author/year	Age (M ± SD) (years) Total sample size	Study setting/study period	Experimental group	Control group	Course	Outcomes
Li 2014 [17]	E: 37.7 ± 3.8,C: 38.2 ± 3.4; 72 (36/36)	RCT; 2009.02-2012.10	FKQJ tablet (6 tablets, tid, po), nitroimidazoles (iv)	Nitroimidazoles (metronidazole, 0.5 g, bid, iv)	2 weeks	①
Zhang 2018 [18]	E: 30.18 ± 2.02,C: 30.26 ± 2.19; 104 (52/52)	RCT; 2016.01-2017.02	FKQJ tablet (6 tablets, tid, po), nitroimidazoles (iv)	Nitroimidazoles (metronidazole, 15 mg, qd, iv)	6 weeks	①⑤
Du 2017 [19]	E: 30.42 ± 2.44,C: 30.71 ± 2.78; 160 (80/80)	RCT; 2016.04-2016.12	FKQJ tablet (6 tablets, tid, po), nitroimidazoles (iv)	Nitroimidazoles (metronidazole, 15 mg, qd, iv)	6 weeks	①⑤
Meng 2018 [20]	E: 30.5 ± 2.5,C: 30.7 ± 2.6; 84 (42/42)	RCT; 2017.05-2018.05	FKQJ tablet (6 tablets, tid, po), nitroimidazoles (iv)	Nitroimidazoles (metronidazole, 15 mg, qd, iv)	6 weeks	①
Chang 2016 [21]	E: 27.34 ± 2.13,C: 27.15 ± 2.25; 80 (40/40)	RCT; 2014.02-2015.10	FKQJ tablet (3 tablets, tid, po), nitroimidazoles (po)	Nitroimidazoles (metronidazole tablet, 2 tablets, tid, po)	6 weeks	①
Fan 2016 [22]	32.57 ± 3.11; 180 (90/90)	RCT; 2014.01-2015.12	FKQJ tablet (6 tablets, tid, po), nitroimidazoles (iv)	Nitroimidazoles (metronidazole, 0.5 g, q8h, iv)	2 weeks	①③
Liu 2017 [23]	E: 32.4 ± 2.6,C: 33.7 ± 2.9; 128 (64/64)	RCT; 2015.10-2017.01	FKQJ tablet (6 tablets, tid, po), nitroimidazoles (po)	Nitroimidazoles (metronidazole tablet, 2 tablets, tid, po)	2 weeks	①③
Guo 2015 [24]	E: 32.5 ± 3.6,C:32.7 ± 3.5; 156 (78/78)	RCT; 2012.02-2014.02	FKQJ tablet (6 tablets, tid, po), nitroimidazoles (iv)	Nitroimidazoles (metronidazole, 15 mg, qd, iv)	6 weeks	①
Shen 2018 [25]	E: 30.57 ± 2.12,C: 30.24 ± 2.13; 78 (39/39)	RCT; 2015.01-2017.10	FKQJ capsules (2 capsules, bid, po), nitroimidazoles (po)	Nitroimidazoles (metronidazole tablet, 2 tablets, tid, po)	2 weeks	①②③
Wang 2018 [26]	E: 31.2 ± 3.4,C: 32.4 ± 3.0; 102 (51/51)	RCT; 2016.04-2017.12	FKQJ tablet (2 tablets, bid, po), nitroimidazoles (po)	Nitroimidazoles (metronidazole tablet, 2 tablets, tid, po)	2 weeks	①②④
Lou 2017 [27]	E: 32.7 ± 4.7,C: 32.1 ± 4.3; 89 (45/44)	RCT; 2013.01-2016.01	FKQJ tablet (6 tablets, tid, po), nitroimidazoles (po)	Nitroimidazoles (metronidazole tablet, 2 tablets, tid, po)	6 weeks	①
Chen 2017 [28]	E: 30.05 ± 4.55,C: 29.51 ± 4.53; 60 (30/30)	RCT; 2015.06-2017.05	FKQJ tablet (6 tablets, tid, po), nitroimidazoles (iv)	Nitroimidazoles (metronidazole, 15 mg, qd, iv)	8 weeks	①
Liu 2019 [29]	E: 30-45,C: 32-47; 78 (39/39)	RCT; 2018.03-2019.03	FKQJ tablet (6 tablets, tid, po), nitroimidazoles (iv)	Nitroimidazoles (metronidazole, 0.5 g, q8d, iv)	4 weeks	①
Zheng 2019 [30]	E: 31.42 ± 0.14,C: 30.14 ± 1.15; 80 (40/40)	RCT; 2016.05-2018.06	FKQJ tablet (6 tablets, tid, po), nitroimidazoles t (po)	Nitroimidazoles (metronidazole tablet, 2 tablets, tid, po)	6 weeks	①
Lin, 2019 [31]	E: 32.5 ± 3.1,C: 31.2 ± 4.5; 60 (30/30)	RCT; 2016.05-2018.05	FKQJ tablet (6 tablets, tid, po), nitroimidazoles t (po, 2 weeks)	Nitroimidazoles (metronidazole tablet, 2 tablets, tid, po, 2 weeks)	6 weeks; antibiotic 2 weeks	①
Zhang 2019 [32]	E:29.5 ± 1.2, C:28.7 ± 2.1; 68(34/34)	RCT; 2017.05-2018.05	FKQJ tablet (6 tablets, tid, po), nitroimidazoles (iv)	Nitroimidazoles (metronidazole, 0.5 g, q8h, iv)	4 weeks	①
Zhang 2017 [33]	E: 30.42 ± 2.65, C: 30.12 ± 2.61; 90 (45/45)	RCT; 2014.01-2015.12	FKQJ capsule (2 capsule, tid, po), clindamycin (iv, 1 week)	Clindamycin (clindamycin, 0.6 g, bid, iv, 1 week)	2 weeks; antibiotic 1 week	①⑤
Yuan, 2015 [34]	E: 31.58 ± 4.85,C: 31.65 ± 4.88; 150 (75/75)	RCT; 2013.05-2015.05	FKQJ tablet (2 tablets, tid, po), clindamycin (iv)	Clindamycin (clindamycin phosphate, 1.5 g, tid, iv)	6 weeks	①
Chen 2019 [35]	E: 5.14 ± 2.75,C: 35.36 ± 2.74; 100 (50/50)	RCT; 2016.07-2018.07	FKQJ capsule (2 capsules, tid, po), cephalosporin (po) + nitroimidazoles (po)	Cephalosporin (cefuroxime axetil tablet, 0.75-1.5 g, tid, po) + nitroimidazoles (tinidazole tablets, 0.5 g, bid, po)	2 weeks	①
Yuan 2012 [36]	E: 34.4 ± 1.8,C: 34.6 ± 1.7; 80(40/40)	RCT; 2012.06-2014.06	FKQJ capsule (2 capsules, tid, po); cephalosporin (iv) + nitroimidazoles (iv)	Cephalosporin (ceftriaxone sodium, 2 g, bid, iv) + nitroimidazoles (metronidazole, 500 mg, bid, iv)	2 weeks	①
He 2017 [37]	E: 33.24 ± 1.39,C: 33.11 ± 2.42; 120 (60/60)	RCT; 2013.08-2016.02	FKQJ tablet (6 tablets, tid, po), quinolones (po)	Quinolones (levofloxacin tablet, 200 tablets, tid, po)	3 weeks	①
Lei 2019 [38]	E: 37.5 ± 3.7,C: 36.8 ± 3.9; 60 (30/30)	RCT; 2016.06-2018.06	FKQJ capsule (2 capsules, tid, po); quinolones (po)	Quinolones (levofloxacin tablet, 200 mg, bid, po)	2 weeks	①④
Deng 2019 [39]	E: 37.5 ± 1.9,C: 36.7 ± 2.0; 348 (174/174)	RCT; 2013.03-2015.02	FKQJ tablet (6 tablets, tid, po), cephalosporin (im) + doxycycline (po)	Cephalosporin (cefoxitin, 2 g, q6h, im) + doxycycline (100 mg, bid, po)	45 days	①

Outcomes: ① markedly effective rate; ② adverse event; ③ improvement of lower abdominal pain; ④ time of abdominal pain disappearance; and ⑤ recurrence rate. Both the tablets and capsules of FKQJ are produced by Zhuzhou Qianjin Pharmaceutical Company Limited; State Drugs Administration (SDA) License Number (GUOYAOZHUNZI): Z43020027 (tablet), Z20020024 (capsule). M, mean; SD, standard deviation; RCT, randomized controlled trial; FKQJ, Fuke Qianjin; E, experimental group; C, control group; qd, once a day; bid, twice a day; tid, three times a day; q6h, every 6 hours; q8h, every 8 hours; po, peros; im, intramuscular; iv, intravenous intravenously.

## Data Availability

All relevant data are within the manuscript and its Supplementary Materials.

## Supplementary Materials

Supplementary Materials S1-PRISMA Checklist.pdf: it contains the PRISMA checklist.
